# The impact of low-frequency, low-force cyclic stretching of human bronchi on airway responsiveness

**DOI:** 10.1186/s12931-016-0464-y

**Published:** 2016-11-14

**Authors:** Morgan Le Guen, Stanislas Grassin-Delyle, Emmanuel Naline, Amparo Buenestado, Marion Brollo, Elisabeth Longchampt, Philippe Kleinmann, Philippe Devillier, Christophe Faisy

**Affiliations:** 1 Laboratory of Research in Respiratory Pharmacology – UPRES EA220, Université Versailles – Saint-Quentin, 11 rue Guillaume Lenoir, F-92150 Suresnes, France; 2 Department of Anesthesiology, Hôpital Foch, Université Versailles – Saint-Quentin, Suresnes, France; 3 Department of Pathology, Hôpital Foch, Suresnes, France; 4 Department of Thoracic Surgery, Centre Médico-Chirurgical du Val d’Or, Saint-Cloud, France

**Keywords:** Cyclic stretching, Airway hyperresponsiveness, Mechanical ventilation, Human bronchus

## Abstract

**Background:**

In vivo, the airways are constantly subjected to oscillatory strain (due to tidal breathing during spontaneous respiration) and (in the event of mechanical ventilation) positive pressure. This exposure is especially problematic for the cartilage-free bronchial tree. The effects of cyclic stretching (other than high-force stretching) have not been extensively characterized. Hence, the objective of the present study was to investigate the functional and transcriptional response of human bronchi to repetitive mechanical stress caused by low-frequency, low-force cyclic stretching.

**Methods:**

After preparation and equilibration in an organ bath, human bronchial rings from 66 thoracic surgery patients were stretched in 1-min cycles of elongation and relaxation over a 60-min period. For each segment, the maximal tension corresponded to 80% of the reference contraction (the response to 3 mM acetylcholine). The impact of cyclic stretching (relative to non-stretched controls) was examined by performing functional assessments (epithelium removal and incubation with sodium channel agonists/antagonists or inhibitors of intracellular pathways), biochemical assays of the organ bath fluid (for detecting the release of pro-inflammatory cytokines), and RT-PCR assays of RNA isolated from tissue samples.

**Results:**

The application of low-force cyclic stretching to human bronchial rings for 60 min resulted in an immediate, significant increase in bronchial basal tone, relative to non-cyclic stretching (4.24 ± 0.16 g vs. 3.28 ± 0.12 g, respectively; *p* < 0.001). This cyclic stimulus also increased the affinity for acetylcholine (−log EC50: 5.67 ± 0.07 vs. 5.32 ± 0.07, respectively; p *p* < 0.001). Removal of airway epithelium and pretreatment with the Rho-kinase inhibitor Y27632 and inward-rectifier K+ or L-type Ca^2+^ channel inhibitors significantly modified the basal tone response. Exposure to L-NAME had opposing effects in all cases. Pro-inflammatory pathways were not involved in the response; cyclic stretching up-regulated the early mRNA expression of *MMP9* only, and was not associated with changes in organ bath levels of pro-inflammatory mediators.

**Conclusion:**

Low-frequency, low-force cyclic stretching of whole human bronchi induced a myogenic response rather than activation of the pro-inflammatory signaling pathways mediated by mechanotransduction.

## Background

Airway tone (defined as the sustained contractile activation of airway smooth muscle (ASM)) and responsiveness are the main functional characteristics of the bronchial compartment of the lung; they provide an adaptive means of adjusting air flow (all along the bronchial tree), the work of breathing or alveolar pressure [1]. ASM responsiveness can be modulated by pharmacological agents, inflammatory agents or external stimulation, such as the cyclic stretching induced by mechanical ventilation [2]. Indeed, the repetitive inflation-deflation caused by positive-pressure ventilation may provoke a specific response by the ASM, which is known to be adversely affected by mechanical strain (especially high-amplitude strain) [3, 4]. Based on the cross-bridge model of contraction, mechanical strain impairs force generation by disrupting actomyosin cross-bridge interactions [5]. There is also evidence to suggest that strain modulates muscle stiffness and force by inducting cytoskeletal remodeling - even at low levels of strain (greater or equal to a lengthening of 3%) [5]. Length adaptation may also contribute to airway hyperresponsiveness (AHR) in asthma [6]. Furthermore, ASM can adapt to the changes in basal tone induced by contractile agonists in a phenomenon known as force adaptation; this may stiffen the ASM and prevent stretching of the ASM layer, thereby limiting bronchoprotection [6].

Although the impact of cyclic stretching has been extensively investigated in isolated alveolar or ASM cells [7–11], data on the putative effects on intact, fresh, human bronchi are scarce (especially for non-stimulated airways) [12]. Some initial data have shown that stretching human bronchi for only 5 min (with a tension that corresponded to an inflation pressure of more than 30 cm H_2_O) altered the crosstalk between airway cells by (i) increasing epithelial leukotriene release via nitric oxide synthase (NOS) activation and (ii) inducing a myogenic response that depends on the Rho-kinase- and WNT-signaling pathways [13]. Moreover, studies in the mouse have shown that mechanical ventilation can trigger the release of pro-inflammatory cytokines by tracheal tissues [14]. Recently, we developed and validated an integrated, ex vivo model for investigating the effects of low-frequency, low-force cyclic stretching on human bronchial tone and responsiveness [15]. The objective of the present study was to investigate the functional and transcriptional responses of intact human bronchi to the repetitive mechanical stress caused by low-frequency, low-force cyclic stretching.

## Methods

### Patients and human bronchus samples

Lung tissue was obtained from macroscopically healthy parts of the lungs of 66 patients (44 males and 22 females; mean ± standard deviation age: 66 ± 10 years) undergoing surgical resection for lung carcinoma. None of the patients had a history of asthma, and all were ex-smokers. The use of resected lung tissue for in vitro experiments was approved by the local institutional review board (*Comité de Protection des Personnes Ile de France VIII*, Boulogne-Billancourt, France). All patients provided their written informed consent to research use of their samples. Tissue dissection was performed by only one experienced operator (EN) in a strictly similar manner, by starting at the main bronchus and then following the bronchial tree to the tissue of interest, i.e. the 4th or 5th level of bronchial division. Bronchial segments were excised from a site as far as possible from the malignant lesion and then immersed in physiologic saline solution (PSS) (NaCl: 119 mM, KCl: 4.7 mM, CaCl_2_: 2.5 mM, KH_2_PO_4_: 1.2 mM, NaHCO_3_: 29 mM, glucose: 11.7 mM) at 4 °C. After removal of adhering lung parenchyma and connective tissue, eight similar bronchial rings (length: 5 mm; inner diameter: 1–2 mm) were prepared as previously described [15, 16].

### Tissue preparation for organ bath studies

Bronchial rings were suspended horizontally on tissue hooks in an organ bath containing 5 ml of PSS (gassed with 95% O_2_, 5% CO_2_) and maintained at 37 °C (pH 7.40). Each preparation was connected to a force displacement transducer (it1, Emka Technologies, Paris, France). Isometric tension was measured and processed with a computerized system running IOX software (v2.4.2) and analyzed with Datanalyst (v2.1.0) software (EMKA technologies, Paris, France). Bronchi were suspended with an initial tension of 1.5 g [15] and equilibrated for 60 min. During the equilibration period, the bath’s PPS was changed after 10, 20 and 30 min. At the end of this period, bronchi were contracted with acetylcholine (3.10^−3^ M) to determine the maximal tone (Emax) developed during contraction in the absence of any treatment or pretreatment. These experiments were run in parallel with appropriate controls (i.e. non-stretched rings or stretched but non-pretreated rings). When required, the epithelium was removed before suspension in the organ bath, as previously described [17].

### Low-frequency, low-force cyclic stretching

**Fig. 1:**
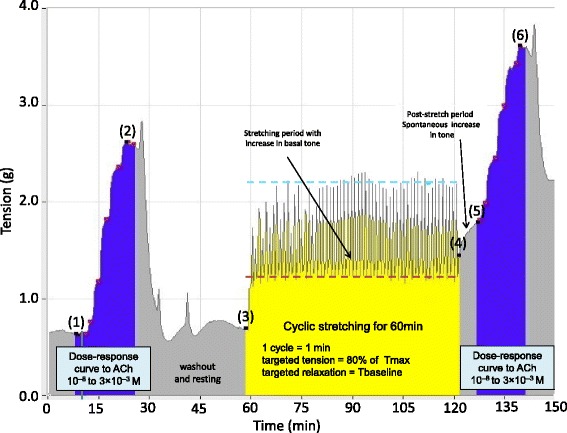
Effect of low-frequency, low-force cyclic stretching on bronchial basal tone and responsiveness to acetylcholine. The illustration is based on a representative experiment. ACh, acetylcholine; CRC, concentration-response curves for ACh; (1), resting basal tone; (2), maximal bronchial contraction in response to 3 mM ACh, before cyclic stretching; (3), pre-stretching bronchial tone; (4), bronchial tone at rest immediately after the end of cyclic stretching; (5), post-stretching bronchial tone within 10 min following the cyclic stretching period; (6) maximal bronchial contraction in response to 3 mM ACh after cyclic stretching. In the control group, bronchial tone did not change significantly between points 4 and 6 (data not shown) (20)

The integrated model for cyclic stretching of isolated human bronchi has already been described in detail and validated [15]. Bronchi were stretched in 1-min cycles over a 60-min period (Fig. 1). Briefly, the stretching cycle consisted of three phases. The first 15-s phase corresponded to an increase in the applied isometric force up to two or three times the basal tone, i.e., an airway inflation pressure of 30 cm H2O [14]; this limits the risk of bronchial damage. This initial tensioning phase was divided into two subphases: a steep increase over 10 s to just below the target value (but without exceeding 80% of Emax), followed by a slower increase to the target value over the last 5 s. These two subphases mimic the increase in insufflated volume during volume-assisted ventilation, and thus limited the risk of over-distension and disruption of the bronchial tissue. The initial tensioning phase was followed by a 15-s phase during which the imposed tension returned to the baseline value, and then a final 30-s phase with no imposed changes in tension [15].

### Functional assays

In order to investigate the putative signaling pathways involved in the stretching-induced response of human bronchi and to characterize the bronchial ring’s responsiveness profile, a cumulative concentration-response curve (CRC) was first established for acetylcholine (ACh: 10 nM to 3 mM). After ACh washout and re-equilibration, samples were pretreated for at least 15 min before the cyclic stretching period. A second CRC for ACh was then established (Fig. 1). The following pretreatment compounds were added to the organ bath: (i) a cyclooxygenase (COX) inhibitor (indomethacin, 1 μM); (ii) a leukotriene Cyst-LT_1_ receptor antagonist (MK476, 1 μM); (iii) a non-specific NOS inhibitor at a high concentration (L-nitroarginine methyl ester (L-NAME), 1 mM); (iv) a L-type calcium channel blocker (nicardipine, 10 μM); (v) a natural blocker of voltage-sensitive Na^+^ channels, with inhibition of action potentials and nerve conduction (tetrodotoxin, 1 μM); (vi) a nonspecific blocker of acid-sensing ion and stretch-activated channels (gadolinium (Gd^3+^), 0.1 mM); (vii) an inhibitor of the inward-rectifier K^+^ channel and large-conductance, calcium-activated potassium channels (tertiapin, 10 μM); (viii) a synthetic compound that acts as an antagonist for the vanilloid transient receptor potential 1 (TRPV1) receptor and inhibits the cold-activated transient receptor potential melastatin 8 (TRPM8) channel (capsazepine, 1 μM); (ix) a protein kinase C (PKC) blocker (calphostin C, 0.1 μM); (x) an inhibitor of the 38-kDa mitogen-activated protein kinase (MAPK) (SB203580, 0.1 μM); and (xi) a selective Rho-kinase (ROCK1 and ROCK2) inhibitor (Y27632, 1 μM). The concentrations of the various drugs were chosen on the basis of the literature data.

### Drugs

ACh, indomethacin, L-NAME, tetrodotoxin, Gd^3+^, nicardipine, capsazepine, and calphostin C were purchased from Sigma-Aldrich (St. Louis, MO, USA), tertiapin was bought from Bachem (Voisin le Bretonneux, France), and SB-203580 was supplied by Calbiochem (San Diego, CA, USA). MK476 came from Merck Sharp & Chibret (Paris, France). Y27632 was purchased from Alexis Biochemicals (San Diego, CA, USA). All drugs (other than indomethacin, nicardipine and SB-203580) were dissolved in distilled water. Indomethacin was dissolved in pure ethanol and then diluted in PSS. Nicardipine and SB-203580 were dissolved in pure ethanol and DMSO and then diluted in PSS. The final amounts of ethanol and DMSO did not alter the responsiveness to ACh [18].

### Analysis of the organ bath fluid

The organ bath levels of mediators involved in (i) the regulation of bronchial basal tone (leukotriene E_4_ (LTE_4_) and prostaglandin E_2_ (PGE_2_)), (ii) the interaction with stretching-induced mechanotransduction (interleukin 8 (IL-8) and tumor necrosis factor-α (TNFα)) and (iii) production of an anti-inflammatory effect (interleukin-10 (IL-10)) were measured immediately before and after cyclic stretching by sampling 250 μl of organ bath fluid from stretched bronchi and their paired, non-stretched controls. Individual fluid samples were assayed using specific ELISAs for stable excretory LTE_4_, PGE_2_, and TNFα (Cayman Chemical Company, Ann Arbor, MI, USA), IL-8 and IL-10 (R&D Systems Inc., Minneapolis, MN, USA). The limit of detection was 25 pg/ml for LTE_4_, 36 pg/ml for PGE_2_, 7.5 pg/ml for IL-8, and 3.9 pg/ml for IL-10 and TNFα. For each assay, all samples were analyzed in a blind manner on the day of the experiment.

### RNA isolation, and reverse transcription quantitative polymerase chain reaction (RT-qPCR array analysis)

To isolate RNA and explore the transcriptional response to cyclic stretching, paired bronchial rings (a control ring and a stretched ring) were immediately immersed in RNAlater reagent (Sigma, St. Louis, MO, USA) after the experiment. The bronchi were frozen and stored at − 80 °C until further use. (RT-qPCR) assays (using TaqMan technology) were performed on bronchial segments that had been crushed and homogenized in TRIzol® reagent immediately after thawing and dissection, using a ball mill TissueLyser LT (Qiagen, Courtaboeuf, France). Total RNAs were extracted from the homogenates using TRIzol® reagent and a method derived from that of Chomczynski and Sacchi [19]. After crushing the preparation in an RNAse-free environment, the amount of RNA extracted was estimated spectrophotometrically at 260 nm (Biowave DNA spectrophotometer; Biochrom, Cambridge, UK), and the purity was assessed with a microfluidic electrophoresis system (RNA Standard Sensitivity kits for Experion®, BioRad, Marnes la Coquette, France). After a DNase treatment step (DNase I, Life Technologies, Carlsbad, CA), 1 μg of total RNA was reverse-transcribed (SuperScript® III First-Strand SuperMix kit, Life Technologies). The resulting cDNA was then used for RT-qPCR experiments in a TaqMan® system (Life Technologies). 20 ng of cDNA was amplified (Gene Expression Master Mix, Life Technologies) in a StepOnePlus thermocycler (Life Technologies). The amplification conditions were as follows: initial denaturation at 95 °C for 10 min, followed by 40 cycles of annealing/extension (95 °C for 15 s, and then 60 °C for 1 min). Fluorescence was measured at the end of each cycle, and the threshold cycle (Ct) of the real-time qPCR was defined as the point at which a fluorescence signal corresponding to the amplification of a PCR product was detectable. The reaction volume was 10 μl. The expression of transcripts of the immediate-early genes of interest (*WNT2*, *WNT3A*, *WNT4*, *WNT5A*, *WNT7B*, *FZD7*, *MAPK1*, *MAPK3*, *MAP3K14*, *MAPK9*, *MAPK14*, *CXCL8*/*IL8*, *COL4A1*, *ELN*, *MMP9*, *LAMC1*, *MYC*, and *CACNA1S*) [13] in the bronchi was analyzed using a specific, predesigned TaqMan® Array (Assay-on Demand, Life Technologies). In order to confirm the extraction of intact cellular mRNA and standardize the quantitative data, three reference genes (those coding for hypoxanthine phosphoribosyltransferase (*HPRT1*), glyceraldehydes-3-phosphate dehydrogenase (*GAPDH*) and β-glucuronidase (*GUSB*)) were amplified simultaneously.

### Data analysis

Data are presented as the mean ± standard error of the mean, together with *n* (the number of experiments). Bronchial tone and contractile responses are expressed in units of tension (g). ACh efficacy (Emax) represents the maximal contraction induced by 3 mM ACh, and ΔEmax (g) represented the difference between Emax (points 2 and 6, Fig. 1) and the basal tone recorded before the CRC (points 1 and 5, Fig. 1). ACh potency (−log EC_50_) represents the negative log of the ACh concentration that produced 50% of Emax. The quantitative data obtained from RT-qPCR experiments correspond to the relative expression (2^−ΔCt^), where ∆C_t_ is the difference between the target gene C_t_ and the mean C_t_ of reference genes. The ELISA results are expressed as the means of duplicate samples. Stretching-induced production of LTE_4_, PGE_2_, IL-8, IL-10 or TNFα is expressed in pg/mg of bronchial tissue, and corresponds to the difference in the amount (concentration × volume) of mediator released into the organ bath immediately before and after cyclic stretching. The results were analyzed using Student’s *t* test for paired and normally distributed data or using a Wilcoxon matched-pairs signed rank test if the data were not normally distributed. The standardized effect size |*d*| for the difference between the means was calculated in order to determine whether the observed effect of each pretreatment was small (|*d*| ≥ 0.20), medium (|*d*| ≥ 0.50) or large (|*d*| ≥ 0.80), according to Cohen’s conventions [20]. The 95% confidence interval (CI) for *d* was calculated as a measure of the uncertainty with regard to the true effect of each pretreatment. A *P* value < 0.05 was considered to be statistically significant. Data analysis and statistical tests were performed using Statistica’99 software, version 5.5, StatSoft, Tulsa, OK, USA).

## Results

### Effect of cyclic stretching on bronchial basal tone and responsiveness

In line with our previous published data [21], we observed a direct effect of cyclic stretching on bronchial tone at rest. Firstly, a spontaneous rise (points 3 to 4 in Fig. 1) was observed immediately after the end of the stretching (1.85 ± 0.06 g, versus 1.20 ± 0.07 g in paired controls; *P* < 0.001, *n* = 66). An additional, post-stretching increase in bronchial tone (points 4 to 5 in Fig. 1) was also observed within 10 min of the end of the cyclic stretching period (2.09 ± 0.07 g, versus 1.21 ± 0.07 g in paired controls, *P* < 0.001, *n* = 66). Cyclic stretching was also associated with an increase in the mean efficacy of ACh (Emax: 4.24 ± 0.16 g in stretched rings, versus 3.28 ± 0.12 g in paired controls, *P* < 0.001, *n* = 66) and the potency of ACh (−log EC_50_: 5.67 ± 0.07 g in stretched rings, versus 5.32 ± 0.07 g in paired controls, *P* < 0.001, *n* = 66).

### Effect of epithelium removal on the impact of cyclic stretching

Epithelium removal was associated with a slight (but not statistically significant) reduction in the immediate post-stretching rise in bronchial tone (Table 1) and a significantly lower late post-stretching rise (Table 2). Moreover, epithelium removal tended to increase the bronchial responsiveness to ACh after cyclic stretching. However, the small standardized size effect and the broad 95% CI indicated a high degree of great uncertainty, and limited our ability to draw conclusions about the true effect of the removal procedure (Tables 3 and 4).

**Table 1:** Effects of pretreatments on the bronchial tone at rest immediately after the end of cyclic stretch

		Cyclic stretch effect on the basal tone during stretching period, difference between control and stretch group		Effect of pretreatment	Effect size pretreatment
	*n*	in absence of pretreatment (g)	in presence of pretreatment (g)	(g)	|d| [95% CI]
Epithelium removal	13	0.76 ± 0.08^*a*^	0.53 ± 0.10^*a*^	−0.23 ± 0.09 ^*d*^	−0.70 [−1.48 − 0.10]
Indomethacin (1 μM)	8	1.14 ± 0.2^*b*^	1.14 ± 0.18^*a*^	0.01 ± 0.10	0.00 [−1.01 − 1.01]
MK476 (1 μM)	7	1.22 ± 0.2^*b*^	0.89 ± 0.14^*a*^	−0.33 ± 0.12^*d*^	−0.68 [−1.70 − 0.44]
L-NAME (1 mM)	14	0.61 ± 0.15^*b*^	1.18 ± 0.15^*a*^	0.57 ± 0.14^*e*^	1.02 [0.20 − 1.77]
Nicardipine (10 μM)	10	0.65 ± 0.12^*a*^	1.03 ± 0.18^*a*^	0.38 ± 0.18	0.78 [−0.16 − 1.66]
Tetrodotoxin (1 μM)	7	1.07 ± 0.19^*b*^	1.29 ± 0.28^*b*^	0.22 ± 0.16	0.35 [−0.73 − 1.38]
Gadolinium (0.1 mM)	8	1.18 ± 0.20^*a*^	1.24 ± 0.15^*a*^	0.06 ± 0.18	0.12 [−0.87 − 1.10]
Tertiapine (10 μM)	9	0.51 ± 0.13^*b*^	−0.74 ± 0.12^*a*^	−1.25 ± 0.17^*f*^	−3.32 [−4.50− −1.85]
Capsazepine (1 μM)	8	1.19 ± 0.14^*a*^	0.92 ± 0.20^*b*^	−0.26 ± 0.23	−0.67 [−1.31− −0.01]
Calphostine C (0.1 μM)	9	1.10 ± 0.23^*a*^	1.12 ± 0.25^*b*^	0.02 ± 0.29	0.03 [−0.90 − 0.95]
SB203580 (0.1 μM)	10	1.11 ± 0.27^*b*^	0.84 ± 0.42	−0.27 ± 0.37	−0.24 [−1.11 − 0.65]
Y27632 (1 μM)	11	1.10 ± 0.25^*b*^	−0.75 ± 0.30^*c*^	−1.85 ± 0.50^*e*^	−2.03 [−2.96– − 0.93]

The difference between pre-stretching basal tone and bronchial tone at rest immediately after the end of stretch (difference between the points 3 and 4, Fig. 1) corresponded to the direct effect of cyclic stretch. Values are means ± standard error of the mean and standardized effect size |d| and its 95% confidence interval [CI] for the difference between means. The observed effect of pretreatment is small (IdI ≥ 0.20), medium (IdI ≥ 0.50), or large (IdI ≥ 0.80) according to the Cohen’s conventions [23]. The 95% CI for |d| consists of the uncertainty around the real effect of pretreatment. ^*a*^ *P* < 0.001, ^*b*^ *P* < 0.01, ^*c*^ *P* < 0.05 stretched vs. paired non-stretched control bronchi. ^*d*^ *P* < 0.05, ^*e*^ *P* < 0.01, ^*f*^ *P* < 0.001 pretreatment vs. no pretreatment

**Table 2:** Effects of pretreatments on the post-stretch response in bronchial tone

		Cyclic stretch effect on post-stretch response, difference between control and stretch group		Effect of pretreatment	Effect size pretreatment
	*n*	in absence of pretreatment (g)	in presence of pretreatment (g)	(g)	|d| [95% CI]
Epithelium removal	13	0.24 ± 0.03^*a*^	0.15 ± 0.03^*a*^	−0.09 ± 0.03^*d*^	−3.00 [−4.01– − 1.80]
Indomethacin (1 μ M)	8	0.36 ± 0.07^*b*^	0.29 ± 0.07^*b*^	−0.07 ± 0.11	−0.37 [−1.34–0.64]
MK476 (1 μM)	7	0.35 ± 0.08^*b*^	0.23 ± 0.06^*c*^	−0.12 ± 0.10	−0.64 [−1.67–0.47]
L-NAME (1 mM)	14	0.29 ± 0.03^*a*^	0.21 ± 0.03^*a*^	−0.08 ± 0.02^*d*^	−0.73 [−1.47–0.06]
Nicardipine (10 μM)	10	0.17 ± 0.03^*a*^	0.12 ± 0.08^*a*^	−0.05 ± 0.07	−0.27 [−1.13–0.63]
Tetrodotoxin (1 μM)	7	0.37 ± 0.08^*b*^	0.29 ± 0.06^*b*^	−0.07 ± 0.10	−0.43 [−1.46–0.66]
Gadolinium (0.1 mM)	8	0.36 ± 0.07^*b*^	0.26 ± 0.03^*a*^	−0.10 ± 0.08	−0.69 [−1.65 − 0.36]
Tertiapine (10 μM)	9	0.23 ± 0.06^*b*^	0.27 ± 0.04^*a*^	0.04 ± 0.08	0.26 [−0.68–1.18]
Capsazepine (1 μM)	8	0.29 ± 0.06^*b*^	0.37 ± 0.05^*a*^	0.08 ± 0.06	0.74 [−0.31–1.71]
Calphostine C (0.1 μM)	9	0.21 ± 0.03^*a*^	0.26 ± 0.02^*a*^	0.05 ± 0.05	0.65 [−0.32–1.57]
SB203580 (0.1 μM)	10	0.29 ± 0.05^*a*^	0.15 ± 0.19	−0.14 ± 0.22	−0.32 [−1.19–0.58]
Y27632 (1 μM)	11	0.28 ± 0.05^*a*^	0.33 ± 0.07^*b*^	0.05 ± 0.08	0.25 [−0.60–1.08]

The post-stretch response in bronchial tone corresponded to the spontaneous post-stretching increase within 10 min following the cyclic stretch period (difference between the points 5 and 4, Fig. 1). Values are means ± standard error of the mean and standardized effect size |d| and its 95% confidence interval [CI] for the difference between means. The observed effect of pretreatment is small (IdI ≥ 0.20), medium (IdI ≥ 0.50), or large (IdI ≥ 0.80) according to the Cohen’s conventions [23]. The 95% CI for |d| consists of the uncertainty around the real effect of pretreatment. ^*a*^ *P* < 0.001, ^*b*^ *P* < 0.01, ^*c*^ *P* < 0.05 stretched vs. paired non-stretched control bronchi. ^*d*^ *P* < 0.05 pretreatment vs. no pretreatment

**Table 3:** Effects of pretreatments on the stretch-induced change in maximal efficacy to acetylcholine

		Cyclic stretch effect on ΔEmax (ACh) difference between control and stretch group		Effect of pretreatment	Effect size pretreatment
	*n*	in absence of pretreatment (g)	in presence of pretreatment (g)	(g)	|d| [95% CI]
Epithelium removal	13	−0.01 ± 0.15	0.27 ± 0.14	0.27 ± 0.12^*a*^	0.54 [−0.26–1.29]
Indomethacin (1 μM)	8	0.12 ± 0.25	0.19 ± 0.11	0.07 ± 0.25	0.25 [−0.75–1.22]
MK476 (1 μM)	7	0.15 ± 0.29	0.51 ± 0.23	0.37 ± 0.28	0.53 [−0.57–1.55]
L-NAME (1 mM)	14	−0.01 ± 0.07	0.26 ± 0.14	0.25 ± 0.11^*a*^	0.66 [−0.12–1.40]
Nicardipine (10 μM)	10	0.25 ± 0.18	1.02 ± 0.44^*b*^	0.76 ± 0.43	0.73 [−0.21–1.60]
Tetrodotoxin (1 μM)	7	0.16 ± 0.25	−0.02 ± 0.12	−0.18 ± 0.30	−0.35 [−1.38–0.73]
Gadolinium (0.1 mM)	8	0.11 ± 0.22	0.39 ± 0.19	0.28 ± 0.28	0.49 [−0.53–1.45]
Tertiapine (10 μM)	9	−0.02 ± 0.08	0.25 ± 0.10^*b*^	0.27 ± 0.18	0.95 [−0.03–1.92]
Capsazepine (1 μM)	8	0.36 ± 0.26	0.29 ± 0.19	−0.07 ± 0.26	−0.11 [−1.08–0.88]
Calphostine C (0.1 μM)	9	0.00 ± 0.28	0.18 ± 0.22	0.18 ± 0.22	0.24 [−0.70–1.15]
SB203580 (0.1 μM)	10	−0.22 ± 0.21	−0.25 ± 0.16	−0.03 ± 0.25	−0.05 [−0.93–0.83]
Y27632 (1 μM)	11	−0.13 ± 0.22	0.21 ± 0.08^*b*^	0.33 ± 0.21	0.63 [−0.25–1.46]

ΔEmax represented the difference in maximal contraction between the pre-stretch (points 1 to 2) and the post-stretch (points 5 to 6) concentration-response curves to ACh (Fig. 1). Values are means ± standard error of the mean and standardized effect size |d| and its 95% confidence interval [CI] for the difference between means. The observed effect of pretreatment is small (IdI ≥ 0.20), medium (IdI ≥ 0.50), or large (IdI ≥ 0.80) according to the Cohen’s conventions [23]. The 95% CI for *d* consists of the uncertainty around the real effect of cyclic stretch. ^*a*^ *P* < 0.05 pretreatment vs. no pretreatment. ^*b*^ *P* < 0.05 stretched vs. paired non-stretched control bronchi. ACh, acetylcholine

**Table 4:** Effects of pretreatments on the stretch-induced change in potency of acetylcholine

		Cyclic stretch effect on Δ (−log EC_50_), difference between control and stretch group		Effect of pretreatment	Effect size pretreatment
	*n*	in absence of pretreatment (log unit)	in presence of pretreatment (log unit)	(log unit)	|d| [95% CI]
Epithelium removal	13	0.48 ± 0.08^*a*^	0.50 ± 0.14^*b*^	0.02 ± 0.13	0.05 [−0.70–0.80]
Indomethacin (1 μM)	8	0.38 ± 0.07^*a*^	0.32 ± 0.13^*b*^	−0.05 ± 0.12	−0.23 [−1.20–0.76]
MK476 (1 μM)	7	0.41 ± 0.07^*c*^	0.26 ± 0.23	−0.15 ± 0.24	−0.34 [−1.37–0.74]
L-NAME (1 mM)	14	0.30 ± 0.07^*a*^	0.44 ± 0.05^*a*^	0.14 ± 0.05	0.54 [−0.23–1.28]
Nicardipine (10 μM)	10	0.57 ± 0.10^*a*^	0.11 ± 0.12	−0.45 ± 0.17^*d*^	−1.26 [−2.16– − 0.25]
Tetrodotoxin (1 μM)	7	0.25 ± 0.05^*c*^	0.38 ± 0.04^*a*^	0.13 ± 0.06	1.12 [−0.07–2.16]
Gadolinium (0.1 mM)	8	0.27 ± 0.05^*b*^	0.49 ± 0.08^*a*^	0.22 ± 0.08^*d*^	1.19 [0.07–2.18]
Tertiapine (10 μM)	9	0.40 ± 0.13^*b*^	0.41 ± 0.09^*c*^	0.03 ± 0.19	0.03 [−0.90–0.95]
Capsazepine (1 μM)	8	0.40 ± 0.11^*c*^	0.54 ± 0.12^*c*^	0.14 ± 0.15	0.41 [−0.60–1.38]
Calphostine C (0.1 μM)	9	0.26 ± 0.04^*a*^	0.24 ± 0.19	−0.01 ± 0.20	−0.04 [−0.80–0.88]
SB203580 (0.1 μM)	10	0.37 ± 0.07^*a*^	0.36 ± 0.10	−0.01 ± 0.13	−0.04 [−0.91–0.84]
Y27632 (1 μM)	11	0.37 ± 0.08^*a*^	0.37 ± 0.16^*b*^	−0.01 ± 0.16	−0.12 [−0.95–0.72]

Δ (−log EC_50_) represented the change in potency between the pre-stretch and the post-stretch concentration-response curves to ACh (Fig. 1). Values are means ± standard error of the mean and standardized effect size |d| and its 95% confidence interval [CI] for the difference between means. The observed effect of pretreatment is small (IdI ≥ 0.20), medium (IdI ≥ 0.50), or large (IdI ≥ 0.80) according to the Cohen’s conventions [23]. The 95% CI for *d* consists of the uncertainty around the real effect of pretreatment. ^*a*^ *P* < 0.001, ^*b*^ *P* < 0.05, ^*c*^ *P* < 0.01 stretched vs. paired non-stretched control bronchi. ^*d*^ *P* < 0.05 pretreatment vs. no pretreatment. ACh, acetylcholine

### Effect of inhibiting the epithelial regulation of smooth muscle contraction on the impact of cyclic stretching

**Fig. 2:**
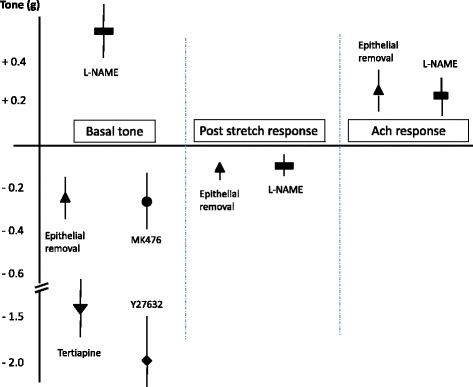
Significant effects of pretreatment in bronchial tone or responsiveness during and following 1 hour of low-force cyclic stretching. Three distinct periods can be seen: during stretch (basal tone), 10 min after cyclic stretch (the post-stretch response) and the maximal tone after a dose–response to acetylcholine (ACh). In all cases, epithelium removal and L-NAME pretreatment were significantly involved

Inhibition of COX with 1 μM indomethacin did not have a significant impact on the stretching-induced increase in bronchial tone and responsiveness to ACh (Tables 1, 2, 3 and 4, and Fig. 2). Likewise, blockade of leukotriene cyst-LT_1_ receptors by MK476 did not have a significant effect on the stretching-induced rises in bronchial tone and responsiveness. In contrast, NOS inhibition by 1 mM L-NAME significantly increased the immediate post-stretching rise in bronchial tone but did not have a marked effect on the late post-stretching rise in bronchial tone or on responsiveness to ACh, as demonstrated by the small standardized effect size and the corresponding 95% CI.

### Effect of drugs that decrease the intracellular Ca^2+^ concentration on the impact of cyclic stretching

Stretching-induced rises in bronchial tone and responsiveness were not significantly modified by pretreatment with 10 μM nicardipine, 1 μM tetrodotoxin, 0.1 mM Gd^3+^ or 1 μM capsazepine (Tables 1, 2, 3, and 4). In contrast, blocking the inward-rectifier K^+^ channel and the calcium-activated large conductance K^+^ channels by applying 10 μM tertiapin significantly decreased the late post-stretching increase in bronchial tone (Table 1). Moreover, blockade of L-type calcium channels by 10 μM nicardipine strongly decreased the effect of cyclic stretching on the potency of ACh, whereas blockade of stretch-activated channels by Gd^3+^ accentuated the effect of stretching (Tables 3 and 4).

### Effect of inhibiting PKC, MAPK and Rho-kinase signaling intracellular pathways on the impact of cyclic stretching

0.1 μM calphostin C or SB203580 had no effect on the stretching-induced rises in bronchial tone and responsiveness to ACh (Tables 1, 2, 3, and 4). Inhibition of Rho-kinases by 1 μM Y27632 significantly decreased the immediate post-stretching increase bronchial tone at rest but did not markedly modify the late post-stretching increase or responsiveness to ACh (Fig. 2).

### Transcript expression and ELISAs of organ-bath fluid samples

**Fig. 3:**
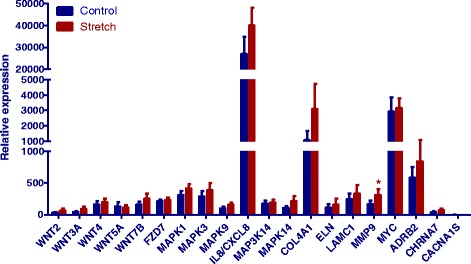
Effect of low-frequency, low-force cyclic stretching on the early mRNA-expression of genes involved in the WNT signaling pathway (*WNT2*, *WNT3A*, *WNT4*, *WNT5A*, *WNT7B* and *FZD7*), the MAPK signaling pathway (*MAPK1* and *MAPK9*), extracellular matrix modulation (*ELN* and *LAMC1*) or actin filament modulation (*COL4A1*), stress or inflammation (*IL8*/*CXCL8*, *MAP3K14*, and *MAPK14*) or apoptosis (*MYC*) or the genes coding for type-L calcium channels (*CACNA1S*), cholinergic receptors (*CHRNA7*) or the β_2−_adrenergic receptor (*ADRB2*) in 9 human bronchial rings. Values were determined with RT-qPCR and are quoted as the relative expression level (2^-ΔCt^), where ∆C_t_ is the difference between the target gene’s C_t_ and the mean C_t_ for simultaneously amplified reference genes. ^*^ *P* < 0.05 for stretched bronchi versus paired, non-stretched control bronchi

**Fig. 4:**
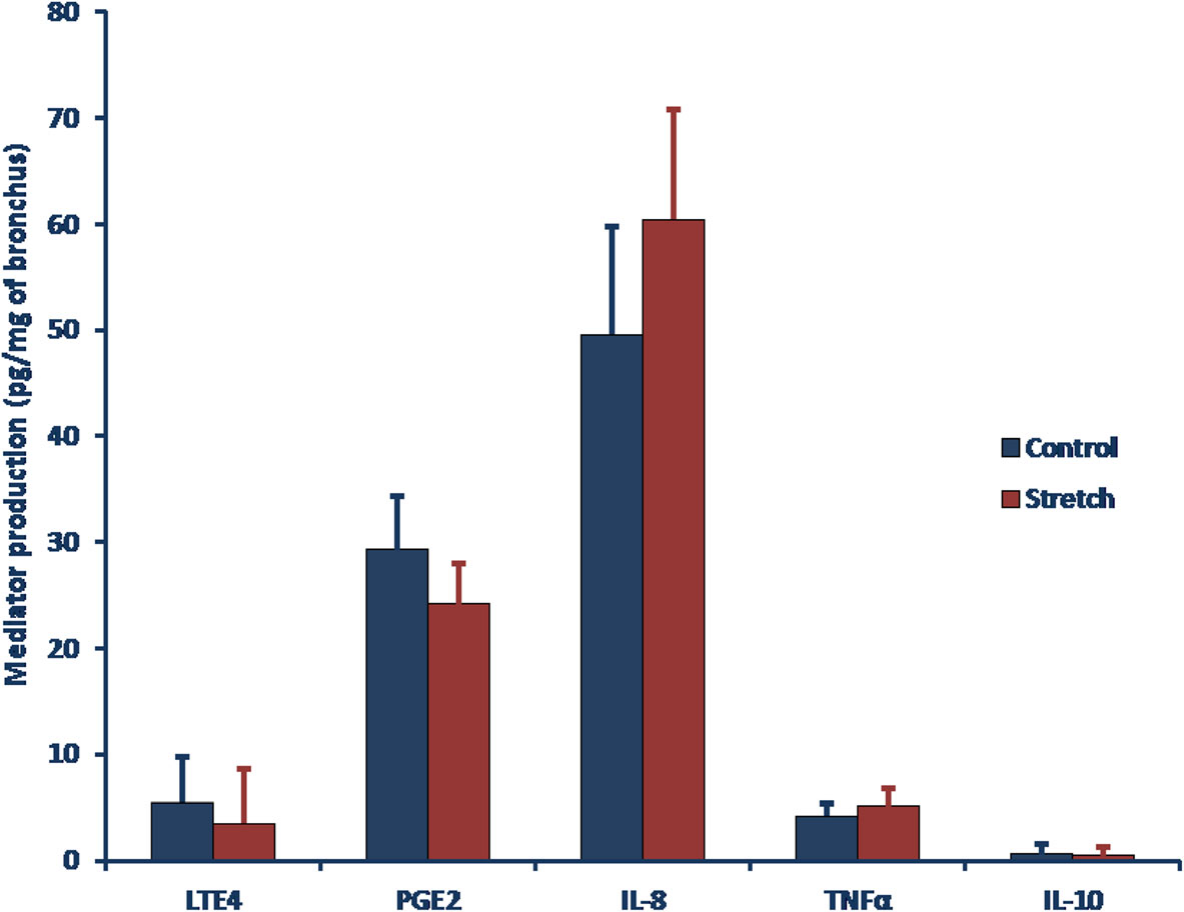
Effect of low-frequency, low-force cyclic stretching on the levels of leukotriene E_4_ (LTE_4_), prostaglandin E_2_ (PGE_2_), and interleukin-8 (IL-8), Tumor necrosis factor-α (TNFα) and interleukin-10 (IL-10) in the organ bath (assessed with ELISAs) in 9 human bronchi. Cyclic stretching (filled bars) was not associated with a significantly change in the production of mediators. Data are quoted as the mean ± SEM

Compared with paired, non-stretched controls, cyclic stretching did not significantly modify the mRNA expression of early genes involved in the WNT signaling pathway (*WNT2*, *WNT3A*, *WNT4*, *WNT5A*, *WNT7B* and *FZD7*), the MAPK signaling pathway (*MAPK1* and *MAPK9*), extracellular matrix modulation (*ELN* and *LAMC1*) or actin filament modulation (*COL4A1*), stress or inflammation (*IL8*/*CXCL8*, *MAP3K14*, and *MAPK14*) or apoptosis (*MYC*) or the genes coding for type-L calcium channels (*CACNA1S*), cholinergic receptors (*CHRNA7*) or the β_2−_adrenergic receptor (*ADRB2*) (Fig. 3). Only the mRNA expression of *MMP9* was significantly greater in stretched rings than in control rings. Moreover, cyclic stretching of the same human bronchi did not significantly change the levels of LTE_4_, PGE_2_, IL-8, IL-10 and TNFα in the organ bath fluid, relative to controls (Fig. 4).

## Discussion

The present study extends our previous observations whereby low-frequency, low-force cyclic stretching of human bronchi was associated with elevated bronchial tone at rest and greater responsiveness to ACh [15]. In the experiments described above, drugs inhibiting pro-inflammatory mediators or the pathways involved in stretching-induced mechanotransduction had no effect on the magnitude of changes in bronchial tone and responsiveness to ACh. Our results suggest that limiting supraphysiologic stress during cyclic stretching may reduce the cascade of signaling events that transduce the external tension from the extracellular matrix to the actin cytoskeleton via the transmembrane integrins; in turn, these signaling events result in the production of contractile mediators, cytokines, growth factors and specific stretch-activated channels, and are coupled with early gene activation [13, 22–25]. This hypothesis is also supported by the absence of an increase in the expression of early genes (other than *MMP9*) and the low production of contractile epithelial mediators in response to low-frequency, low-force cyclic stretching.

The stretching-induced rise in bronchial tone observed in the present study was modulated by airway epithelium removal, NOS inhibition, blockage of the inward-rectifier K^+^ channel, and inhibition of Rho-kinases - suggesting that cyclic stretching may impair the epithelial regulation of ASM tone, alter ASM membrane depolarization, and stimulate the Rho-kinase pathways. Moreover, the stretching-induced increase in ACh potency involved the intracellular Ca^2+^ concentration via the voltage-dependent L-type Ca^2+^ channels but not via the stretch-activated channels, since nicardipine (but not Gd^3+^) diminished the effect of stretching. Overall, our results indicate that the impact of low-frequency, low-force cyclic stretching on the bronchial responsiveness to ACh might be triggered by the Ca^2+^-dependent sensitization of ASM contractile filaments.

In vivo, the intrinsic tone of the human bronchus is modulated by both nervous stimulation and the balance between the contractile mediators (leukotrienes and histamine) and bronchodilatory mediators (PGE2 and nitric oxide) secreted by the airway epithelium [26]. In previously published work on a model of a single, constant stretch of human bronchial rings with a tension of 2.5 times the basal tone, the rise in bronchial tone following stretching was associated with increased activity of the epithelial Ca2 + −independent NOS-2 and the release of LTE4 [13]. In the present study, none of the contractile mediators of mechanotransduction was involved in the stretching-induced rise in bronchial tone. The disparities between the present results and those recorded previously in our model of a single, constant stretch might be explained by differences in the tension generated by the experimental device or by differences in the stretching duration. Therefore, the present findings suggest that airway epithelial cells and pathways with short activation times (such as cellular Ca^2+^ influx via membrane depolarization and Rho-kinase activation) contribute to the observed differences between a single stretch and cyclic stretching, since epithelium removal and pretreatment with tertiapin or Y27632 dampened the stretching-induced rise in bronchial tone. Stretch-activated receptors (which are located along the bronchial tree and may increase airway tone through C-fiber nerve stimulation when exposed to repetitive strain [27]) are unlikely to be involved because pretreatment with tetrodotoxin (an inhibitor of nerve conduction) did not change the stretching-induced rise in bronchial tone. Similarly, the involvement of stretch-activated channels or transient receptor potential ion channels (antagonized by Gd^3+^ and capsazepine, respectively) seems unlikely. In agreement with our previous results on a single, non-cyclic stretch, our present data confirmed the involvement of Rho-kinases in the rise in bronchial tone elicited by cyclic stretching. However, Rho-kinases did not appear to be activated by the early genes that interact with Wnt-signaling pathways, as suggested by our previous experiments in a model of supraphysiologic stretching [13]. These important observations suggest that limiting airway stretching to the low tensions experienced under physiological conditions might reduce the harmful effects of mechanical ventilation on the bronchial compartment. Moreover, it has been shown that the cessation of insufflation with tidal volumes results in further bronchoconstriction within 1 min (regardless of the presence or absence of deep inspiration) [28], suggesting that further airway constriction was solely due to the removal of tidal oscillations via two separate effects (a reduction in the mean load on the ASM and the disappearance of oscillatory load). Accordingly, integrity of the epithelium and the maintenance of a constant level of force on the ASM appear to be essential for preventing constriction. The low-force strain was enough to increase the stiffness of the bronchial ring during our dynamic and cyclic stretching, as suggested by Norris et al.. The latter researchers described a biphasic ASM response to an isovelocity stretch; the first phase concerned the actomyosin interaction and the second phase was dependent on the ASM’s level of activation [5]. The absence of a pre-inflammatory bronchial state is another possible explanation for the absence of a rise in levels of mediators. Previous studies have shown that a moderate tidal volume during mechanical ventilation did not per se cause extensive lung injury in normal lung but did increase inflammatory responses in pre-injured lungs [29]. Although most studies have not observed an increase in the levels of cytokines such as TNF-α and MIP-2 during mechanical ventilation with moderate tidal volumes, a more pronounced increase in these cytokines was observed when mechanical ventilation was combined with exposure to other harmful factors (such as lipopolysaccharide) [30]. In accordance with these studies, our present results show that moderate tidal stretch did not induce the production of IL-8, IL-6 or TNF-α. The contractile apparatus in ASM might be the key effector in the impact of cyclic stretching on bronchial tone. Indeed, bronchial tone depends on the locked actin-myosin bridges, which in turn are related to electromechanical coupling in ASM [31]. The airway inflation-deflation caused by breathing or deep inspiration exerts several different effects on the ASM’s contractile apparatus [32]. Cyclic stretching may alter the initial equilibrium by enhancing polymerization of actin filaments via conformational changes, intracytosolic calcium transfer and activation of the Rho-kinase pathway [33]. The post-stretching increase in bronchial tone may result from ASM stiffness or impaired bronchial relaxation. Indeed, we found that the increase in bronchial tone was abolished by epithelium removal and potentiated by the inhibition of NOS - suggesting that stretching perturbs the epithelial regulation of ASM [34]. A recent study of isolated ASM strips by Ansell et al. demonstrated a significant difference in the maximal contraction induced by electrical field stimulation after sustained changes in length [35].

We found that cyclic stretching increased the potency of ACh more than its efficacy. This phenomenon might be mediated by Ca^2+^ sensitization or by the recruitment of contractile units within ASM. However, we also found that drugs that decrease the intracellular Ca^2+^ concentration or inhibit the intracellular pathways involved in Ca^2+^ sensitization (MAPK, PKC, and Rho-kinases) did not prevent the hyperresponsiveness induced by cyclic stretching; this finding indicates that the response to stretching was myogenic and not related to Ca^2+^ sensitization. In this respect, it is known that force oscillations cause rearrangements in the contractile apparatus, which can then generate a force that exceeds the pre-stretching level [36–38]. The plasticity of ASM and the latter’s structural adaptation to mechanical stress may provide an alternative means of regulating bronchial responsiveness [21]. Our results might also be explained (at least in part) by the difference between the respective effects of “stretch-compress” and “compress-stretch” actions on ASM. In the case of a “stretch-compress” action (as in the present study), the muscle tension rises sharply at the start of the stimulus. This includes the response of the bronchial tree embedded within the elastic component of the bronchial structure [39].

It has been shown that cyclic stretching during mechanical ventilation with a high tidal volume induces the activation of immediate-early genes (especially transcription factors, stress proteins, and inflammatory mediators) in the alveolar compartment [40, 41]. Here, we found that low-frequency, low-force cyclic stretching only increased the expression of *MMP9* mRNA in the bronchial compartment. *MMP9* is up-regulated by *IL*-*8* and encodes an enzyme that degrades type IV and V collagens (both of which are components of the airway sub-epithelial basement membrane) and promotes the relaxation of smooth muscle [42]. Lastly, the results of our transcriptional analysis suggest that limiting airway inflation may reduce the bronchial impact of mechanical ventilation.

Our study had strengths and limitations. One notable strength relates to the use of a tension corresponding to each bronchial segment’s intrinsic properties. Stretching the human bronchial rings with excessive tension would doubtless have generated different results. A second strength relates to the use of freshly human isolated bronchi. This is of real importance because there are interspecies differences in the modulation of airway tone and responsiveness [23]. However, we cannot rule out the possibility that bronchi from patients undergoing thoracic surgery may have been stretched during the surgical procedure. Due to ethical considerations, surgical specimens from healthy non-smokers are extremely scarce and so specific analysis of this material could not be performed. Moreover, further studies should focus the effects of cyclic stretch at higher frequency. Another study limitation relates to the fact that we did not measure the ring’s diameter during cyclic stretching; this would have enabled us to model the induced strain. Furthermore, the short time between stretching and mRNA extraction may have restricted our investigation to very early genes that were up- or down-regulated by cyclic stretching. Lastly, as is the case with other isolated organ models, our experiments were performed under non-aerated conditions and in the absence of surfactant, which could have promoted bronchial narrowing.

## Conclusions

Taken as a whole, our results indicate that low-frequency, low-force cyclic stretching of human bronchi increases bronchial tone and responsiveness primarily via a myogenic response mediated by adaptive changes in the physical state of ASM. Although pro-inflammatory components (in either the cytosol or the organ bath) did not seem to be involved in this response to low-frequency, low-force cyclic stretching, interaction between epithelium and ASM emerged as a major factor. Lastly, our findings in an experimental model of cyclic stretching provide insights into the nature of ventilator-induced lung damage.

## Abbreviations

ACh: Acetylcholine
ADRB2: The gene coding for the β_2_-adrenergic receptor
ASM: Airway smooth muscle
CACNA1S: The gene coding for the voltage-dependent L-type calcium channel subunit α-1S
CHRNA7: The gene coding for the acetylcholine receptor
COL4A1: The gene coding for collagen, type IV, α_1_
COX: Cyclooxygenase
CRC: Concentration-response curve
ELN: The gene coding for elastin
FZD: The gene coding for frizzled family receptor
IL: Interleukin
IL8/CXCL8: The gene coding for IL-8
L-NAME: L-nitroarginine methyl ester
LT: Leukotriene
MAP3K14: The gene coding for mitogen-activated protein kinase kinase kinase 14
MAPK: Mitogen-activated protein kinase
MAPK14: The gene coding for mitogen-activated protein kinase 14
MMP9: The gene coding for matrix metallopeptidase 9
MYC: The gene coding for transcription factor p64
NOS: Nitric oxide synthase
PG: Prostaglandin
PKC: Protein kinase C
PSS: Physiologic saline solution
RT-qPCR: Reverse transcription quantitative polymerase chain reaction
TNFα: Tumor necrosis factor-α
TRPM8: Cold-activated transient receptor potential melastatin 8 channel
TRPV1: Vanilloid transient receptor potential 1 receptor
WNT: Wingless-related integration site, family of signaling protein
